# Diet rich in high glucoraphanin broccoli reduces plasma LDL cholesterol: Evidence from randomised controlled trials

**DOI:** 10.1002/mnfr.201400863

**Published:** 2015-04-07

**Authors:** Charlotte N Armah, Christos Derdemezis, Maria H Traka, Jack R Dainty, Joanne F Doleman, Shikha Saha, Wing Leung, John F Potter, Julie A Lovegrove, Richard F Mithen

**Affiliations:** 1 Food and Health Programme, Institute of Food ResearchNorwich, UK; 2 Hugh Sinclair Unit of Human Nutrition and Institute for Cardiovascular and Metabolic Research, University of ReadingWhiteknights, Reading, UK; 3 Faculty of Medicine and Health Sciences, University of East AngliaNorwich, UK

**Keywords:** Broccoli, Cholesterol, Glucoraphanin, Sulforaphane

## Abstract

**Scope:**

Cruciferous-rich diets have been associated with reduction in plasma LDL-cholesterol (LDL-C), which may be due to the action of isothiocyanates derived from glucosinolates that accumulate in these vegetables. This study tests the hypothesis that a diet rich in high glucoraphanin (HG) broccoli will reduce plasma LDL-C.

**Methods and results:**

One hundred and thirty volunteers were recruited to two independent double-blind, randomly allocated parallel dietary intervention studies, and were assigned to consume either 400 g standard broccoli or 400 g HG broccoli per week for 12 weeks. Plasma lipids were quantified before and after the intervention. In study 1 (37 volunteers), the HG broccoli diet reduced plasma LDL-C by 7.1% (95% CI: –1.8%, –12.3%, *p* = 0.011), whereas standard broccoli reduced LDL-C by 1.8% (95% CI +3.9%, –7.5%, ns). In study 2 (93 volunteers), the HG broccoli diet resulted in a reduction of 5.1% (95% CI: –2.1%, –8.1%, *p* = 0.001), whereas standard broccoli reduced LDL-C by 2.5% (95% CI: +0.8%, –5.7%, ns). When data from the two studies were combined the reduction in LDL-C by the HG broccoli was significantly greater than standard broccoli (*p* = 0.031).

**Conclusion:**

Evidence from two independent human studies indicates that consumption of high glucoraphanin broccoli significantly reduces plasma LDL-C.

## 1 Introduction

Human intervention studies have provided evidence that diets rich in cruciferous vegetables may modify plasma lipid and cholesterol profiles [1–5]. At a population level, a 1% reduction in LDL-cholesterol (LDL-C) has been associated with a 1–2% reduction in risk of coronary artery disease [6]. Recent studies have also highlighted the requirement for cholesterol by cancer cells [7], which have led to a growing interest in the use of statins as cancer therapeutic agents [8]. Thus, a reduction in LDL-C by cruciferous vegetables may contribute to the putative health promoting properties of these vegetables.

We have previously described the development of broccoli cultivars that have enhanced expression of the Myb28 transcription factor due to introgression of a novel Myb28 allele from the wild species *Brassica villosa*. The presence of a single Myb28 *villosa* allele results in a threefold greater accumulation of 4-methylsulphinylbutyl glucosinolate [‘glucoraphanin’], the precursor of the potent nuclear factor [erythroid-derived 2]-like 2 (nrf2) inducer, sulforaphane, compared to that within standard broccoli cultivars [9]. As these ‘high glucoraphanin’ (HG) cultivars are identical in appearance and other nutritional components to standard broccoli cultivars [9] they enable blinded human intervention studies to explore the role of glucoraphanin on biomarkers of health. We have previously reported that consumption of HG broccoli results in enhanced nrf2-mediated transcription in human gastric biopsies compared to consumption of standard broccoli [10], and that consumption of 400 g HG broccoli each week over a 12 week period affected plasma metabolites in a manner consistent with improved integration of fatty acid β-oxidation and TCA cycle activity within mitochondria, probably due to modulation of cellular redox status [11]. However, while we observed significant changes in plasma lipid and steroid metabolites in this previous study, the reduction in LDL-C by the HG broccoli was not significantly greater than that observed with the standard broccoli [11]. Here, we provide more detailed analyses of the changes in plasma lipids in this first study, and report a second larger dietary intervention study of near identical design to test the hypothesis that consumption of 400 g/week of HG broccoli for 12 weeks would significantly reduce plasma LDL–C compared with standard broccoli. We additionally investigate whether there is any association between the broccoli consumption and genotype at glutathione transferase M1(GSTM1), poly[A] polymerase gamma (PAPOLG) and apolipoprotein E (APOE) loci all of which have been associated with diet-gene interactions [11–13].

## 2 Materials and methods

### 2.1 Study 1

The study design and details of recruitment has been previously reported [11]. Briefly, male and female volunteers aged 50–77 years were recruited from within a 40 mile radius of the city of Norwich, United Kingdom who had a 10 year cardiovascular risk profile of between 10 and 20%, estimated using the JBS2 CVD risk assessor [14]. Volunteers were required to consume 400 g of HG broccoli or 400 g of a standard broccoli [cultivar Ironman] for 12 weeks, without any other dietary restrictions. The two broccoli genotypes, which looked identical, were coded by a third party, and their identity was not known to the study volunteers and investigators until completion of data collection. The HG broccoli contained 21.6 ± 1.60 μmol/g dry weight glucoraphanin (4-methylsulphinylbutyl glucosinolate) and 4.5 ± 0.34 μmol/g dry weight glucoiberin (3-methylsulphinylpropyl glucosinolate), whereas the standard broccoli contained 6.9 ± 0.44 μmol/g dry weight glucoraphanin and 0.7 ± 0.33 μmol/g dry weight glucoiberin. There were no differences in the concentrations of indole glucosinolate between the two types of broccoli. The broccoli was grown and harvested with standard agronomic practices. Heads were floreted, blanched and frozen (–20°C) with standard commercial procedures [11]. Total cholesterol (TC), high density lipoprotein-cholesterol (HDL-C) and triacylglycerol (TAG) were analysed in fasted blood samples immediately before and after the dietary intervention, as previously described [11].

### 2.2 Study 2

#### 2.2.1 Participants

Men and women were enrolled into the second study between August 2012 and October 2013. Participants with a 10 year cardiovascular risk profile of between 10 and 30% estimated using the JBS2 CVD risk assessor [14] were eligible to participate in the study. Those who were aged <50 years, had suffered a stroke, myocardial infarction or transient ischemic attack, had a BMI of <20 or >40, a diagnosis of diabetes or a fasting glucose level of >6 mmol/L, a blood pressure > 160/90 mmHg, a fasting cholesterol > 8 mmol/L, were on any form of medication known to affect the cardiovascular system or who were diagnosed with chronic kidney disease were excluded. Those that satisfied the above criteria, ascertained at an eligibility screening appointment, entered the study after informed consent was obtained. Participants were asked to continue any permitted prescribed medication for the duration of the trial and to inform the investigators of any changes in medication. Participants on certain supplements (including glucosamine and cod liver oil) were asked to discontinue use for at least five weeks prior to starting the intervention. The study was approved by the Institute of Food Research's Human Research Governance Committee (Ref: IFR01/2012), Norfolk Research Ethics Committee (Ref: 12/EE/0313), NHS Norfolk & Waveney Research Governance Committee (Ref: 2012GP17 (104550)), The University of Reading Research Ethics Committee (Ref: 12/32) and as the study was adopted onto the National Institute for Health Research Clinical Research Network [NIHR CRN] Portfolio, approval was also obtained from the NIHR coordinated System for Gaining NHS Permission (Ref: 12635). The study is registered at http://clinicaltrials.gov (registration ID no. NCT01929564) and conducted according to the Declaration of Helsinki.

#### 2.2.2 Sample size

**Figure 1:**
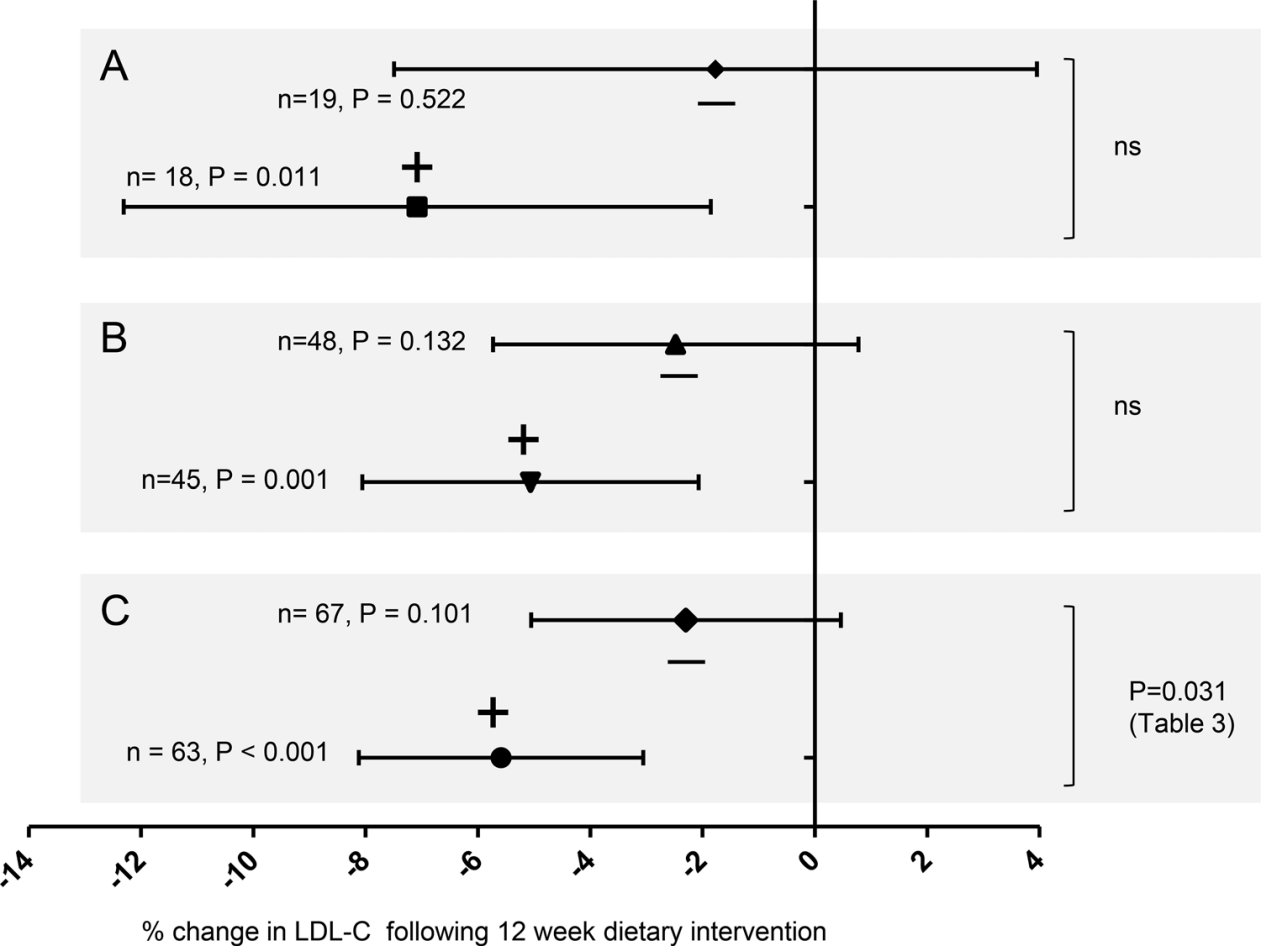
Change in LDL-C following dietary intervention with standard broccoli [-] or high glucoraphanin broccoli [+]. Each panel shows the mean and 95% CI for the change in LDL-C as evident from paired *t*-tests between the baseline and post intervention LDL-C for each individual within the two dietary arms, and an ANOVA for the difference based upon a general linear model [Table 3]. (A) Study 1 (B) Study 2. (C) Combined data from Study 1 and Study 2.

Sample size was estimated based on the following considerations. The previous study that evaluated the effects of consumption of 400 g/week of HG broccoli diet on lipid profile, showed that there was no statistically different effect of the HG broccoli diet on TC, LDL-C or HDL-C compared to standard broccoli [11], but suggested a difference may be apparent with a larger study (Fig. 1A). The data on LDL-C estimated a minimum sample size of 48 participants per arm for this current study, thus a total of 96 participants was predicted to be required. This calculation was based on four assumptions: [a] The mean baseline concentration of LDL-C of the total recruited population would equal 3.77 mmol/L and the interparticipant standard deviation would equal 0.39 mmol/L, [b] the change due to the HG broccoli would be 0.20 mmol/L, while the mean of the standard broccoli group will remain constant at 3.77 mmol/L, [c] a significance level of 0.05 for 80% power and [d] the study is one-tailed test.

#### 2.2.3 Intervention

Male and female participants aged 50–76 years were recruited from within a 40 mile radius of the Institute of Food Research in Norfolk and the University of Reading in Berkshire, United Kingdom. A combination of participant databases, GP surgeries, distributed flyers/poster and word of mouth were used for recruitment. Participants were recruited during the period of August 2012 and October 2013 on the basis of a fasted [≥8 h] screening blood and urine sample, the completion of an eligibility questionnaire and their risk score calculated using the JBS2 algorithm [12]. A double-blind, 12 week randomized two-arm parallel study was then undertaken with men and women who after screening were judged to be at mild or moderate risk of CV, based upon the JBS2 risk estimator (10–30% risk score), as described above. Participants were required to consume 400 g of HG broccoli or 400 g of a standard broccoli cultivar as part of their habitual diet for 12 weeks, without any other dietary restrictions. The two broccoli genotypes were coded by a third party and their identity was not known to the study investigators or participants until completion of data collection and analyses. Participants were randomised centrally as they were recruited by a minimisation program [minim] [15] stratified by age, sex, BMI, smoking status and plasma LDL-C concentrations, to minimise any differences between the treatment groups over the two sites.

The development and phenotypic analysis of HG broccoli has been reported previously [9]. Briefly, HG broccoli contains a Myb28 transcription factor, introgressed by conventional breeding from the wild species *B. villosa*, which results in enhanced assimilation of sulphate and channelling of the additional sulphur to glucoraphanin [9]. Previous studies have reported the pharmacokinetics of sulforaphane metabolism in human volunteers following consumption of standard and HG broccoli [16]. The standard broccoli [cultivar Parthenon] and the HG broccoli (hybrid 1199) were grown and harvested by Seminis in Boston Lincolnshire, with the use of standard agronomic procedures. Heads were harvested, floreted, blanched and frozen (–20°C) as described previously using standard commercial practice [11]. Following processing, the HG broccoli contained 24.83 ± 1.19 μmol/g dry weight glucoraphanin and 5.67 ± 0.40 μmol/g dry weight glucoiberin, whereas the standard broccoli contained 9.47 ± 0.25 μmol/g dry weight glucoraphanin and 1.77 ± 0.12 μmol/g dry weight glucoiberin. There were no differences in the concentrations of indole glucosinolate between the two broccoli cultivars. The frozen broccoli was delivered to the participants in their homes every 3 to 6 weeks, or was collected by the participants from their nearest study centre. Participants prepared the broccoli cultivars from frozen by steaming for up to 5 min, having been provided with a steamer, written instructions and a cooking demonstration.

This study required 96 participants in total recruited over both sites with an equal split for the standard and HG broccoli group. Participants were requested to avoided caffeine for 24 h, excessive exercise for 12 h and fast for at least 8 h prior to their study visit at their local centre [either Human Nutrition Unit, Institute of Food Research Norwich or Hugh Sinclair Unit of Human Nutrition, University of Reading]. Participants were asked to attend two study visits pre- and post-intervention. On arrival at the unit a blood sample was taken by venepuncture, following this an ambulatory blood pressure monitor (Spacelabs 90207, Spacelabs Healthcare, USA) was used to measure the participants’ systolic and diastolic blood pressure every ten minutes for an hour while they rested supine in a quiet room.

#### 2.2.4 Plasma biomarkers and statistical analyses

Fasted blood samples were centrifuged at 1800 × *g* for 15 min at 20°C (for serum) and 4°C (for plasma), and stored at −80°C. Total cholesterol, high density lipoprotein-cholesterol and triacylglycerol were quantified using an autoanalyzer [reagents and analyzer: Instrumentation Laboratory Ltd., Warrington, UK] at the University of Reading. The concentration of LDL was calculated using the Friedewald equation [17]. Paired *t*-tests with 95% confidence intervals were used to analyse the change in LDL-C, TC, HDL-C and TAG within the two arms of each of the studies, and ANOVA-General Linear Model was used to compare the changes between arms in LDL-C, TC, HDL-C and TAG and to quantify the effect of study, recruitment centre, sex and GSTM1, PAPOLG and APOE genotypes.

#### 2.2.5 Dietary intake analysis

Participants completed weekly tick sheets during the 12-week intervention period to identify when the portions of the broccoli were eaten. Every four weeks, participants were contacted by telephone and asked about adherence to the diet. A 7-day estimated food intake diet diary was completed by participants at baseline and after 10 weeks using household measures as an indication of portion size. Participants were instructed by study scientists how to assess portion size and complete the diaries. On completion, the diaries were checked for completeness with quantities verified with the participant, prior to being sent to the University of Reading for analysis. Food intake information from the diaries was assessed by Diet Plan6 (Forest Software, West Sussex) and analysed for differences in nutrient composition between the intervention groups at baseline and 10 weeks after the start of the intervention with ANOVA.

### 2.3 SNP genotype analysis

Genomic DNA samples were isolated from the blood of volunteers by using the QIAGEN DNeasy Blood & Tissue kit according to manufacturer's instructions. DNA samples were genotyped for the SNPs rs74837985 (GSTM1, #C_44202997_20), rs28459296 (PAPOLG, #C_62181387_10), rs7412 (APOE, #C_904973_10) and rs429358 (APOE, #C_3084793_20) using predesigned TaqMan® SNP Genotyping Assays (Life Technologies) on an Applied Biosystems® StepOnePlus™ Real-Time PCR System. APOE alleles 2–4 were determined from the genotype combination of rs7412 and rs429358 [18].

## 3 Results

### 3.1 Study 1

Participant recruitment and dietary intake analyses have been previously reported [11]. Pre- and post-plasma lipid data were obtained from 19 volunteers on the standard broccoli diet and 18 volunteers on the HG broccoli diet.

### 3.2 Study 2—Participant recruitment

Of the 510 participants who attended introductory talks across both study sites, 424 were screened and 107 were recruited onto the study. Of these, eight withdrew for personal reasons, one withdrew due to a protracted illness, one was prescribed blood pressure medication by their clinician and was withdrawn, one developed an allergy to the ambulatory blood pressure cuffs and we were unable to obtain blood from one. In total 93 participants completed the study of which 45 participants were allocated to HG broccoli and 48 to standard broccoli (Supporting Information Fig. 1, Table 1). All women were either post- or peri-menopausal. All participants completed the weekly tick sheet, all of which were 100% compliant with the dietary intervention.

**Table 1:** Baseline characteristics of volunteers for Study 2

	High glucoraphanin broccoli		Standard broccoli	
	Male (*n* = “28)	Female (*n* = 19)	Male (*n* = 27)	Female (*n* = 21)
Age (y)	60.2 ± 6.38a)	63.0 ± 5.72	59.8 ± 5.49	63.5 ± 7.09
BMI (kg/m2)	26.7 ± 3.43	26.0 ± 4.70	27.5 ± 3.67	26.3 ± 4.20
Systolic BP (mmHg)	128.1 ± 14.80	128.4 ± 12.9	126.3 ± 11.24	134.1 ± 10.84
Diastolic BP (mmHg)	83.1 ± 9.87	76.9 ± 10.20	81.6 ± 7.51	78.9 ± 6.10
Weight (kg)	83.4 ± 13.06	68.9 ± 10.14	86.5 ± 12.35	68.0± 12.25
Waist circumference (cm)	98.7 ± 11.20	87.6 ± 11.73	100.5 ± 8.66	87.2 ± 11.50
Hip circumference (cm)	105.5 ± 6.25	107.0± 8.66	106.8 ± 6.95	105.3 ± 10.17
Total cholesterol (mmol/L)	5.9 ± 0.86	6.7 ± 0.71	5.8 ± 0.96	6.7 ± 0.90
LDL cholesterol (mmol/L)	4.2 ± 0.71	4.6 ± 0.77	4.1 ± 0.79	4.7 ± 0.80
HDL cholesterol (mmol/L)	1.4 ± 0.28	1.8 ± 0.32	1.4 ± 0.31	1.7 ± 0.29
Triglycerides (mmol/L)	1.6 ± 0.69	1.1 ± 0.32	1.5±0.57	1.2 ± 0.30
Glucose (mmol/L)	5.7 ± 0.47	5.7 ± 0.46	5.7 ± 0.43	5.7 ± 0.48
JBS2 CVD risk score (%)	17.5 ± 5.85	14.4 ± 4.89	17.1 ± 5.54	12.5 ± 2.52

a) All values are mean ± SD.

#### 3.2.1 Study 2—Dietary intake analysis

There were no significant differences between dietary components either between the two arms of the study or between the two dietary assessments for each individual except for % energy (E) carbohydrate which was higher after the HG broccoli compared with the standard broccoli (*p* = 0.003), although there were no differences in the %E sugars or %E starch (Supporting Information Table 1).

### 3.3 SNPs

The frequency of the *APOE2, APOE3* and *APOE4* alleles were 6.7, 73.9 and 19.3% that are not significantly different from that previously reported in Caucasian population (χ^2^ = 2.84, *p* = 0.242) [13]. At baseline, individuals with the E4/E4 genotype had significantly greater levels of TC and LDL-C than those individuals with E2/E3 genotype (Table 2). The frequency of the *PAPOLG A* and G alleles and the *GSTM1* null genotype were similar to that previously reported [11]. There was no association between *PAPOLG* and *GSTM1* genotype and plasma TC, LDL-C or TAG levels.

**Table 2:** APOE genotype frequency and baseline plasma cholesterol and triacylglycerol levels

	Frequency (%)	Total C	LDL-C	HDL-C	Triacylglycerol
E2/E3	11	5.1 ± 1.04a)	3.5 ± 0.87	1.3 ± 0.25	1.5 ± 0.63
E2/E4	2	5.3 ± 0.86	3.5 ± 0.21	1.5 ± 0.66	1.4 ± 0.43
E3/E3	55	6.2 ± 0.86	4.3 ± 0.75	1.6 ± 0.31	1.2 ± 0.43
E3/E4	28	6.2 ± 0.97	4.3 ± 0.75	1.6 ± 0.41	1.4 ± 0.64
E4/E4	4	7.0 ± 0.91b)	5.0 ± 0.87b)	1.7 ± 0.22	1.3 ± 0.65

a) All values are means ± SD.

b) E4/E4 genotypes have significantly greater Total-C and LDL-C than E2/E3 genotypes [*p* = 0.001, ANOVA].

### 3.4 Plasma lipid and cholesterol profiles

In study 1, volunteers consuming the HG broccoli had significant reduction in plasma LDL-C compared to their own baseline level (Fig. 1A), but with no significant changes in HDL-C, TC or TAG (Supporting Information Figs. 2–4). Near identical results were obtained in study 2, in which volunteers consuming the HG broccoli had significant reductions in LDL-C (Fig. 1B). When data from the two studies were combined, the reduction in LDL-C by the HG broccoli was significantly greater (*p* = 0.031) than that of the standard broccoli (Fig. 1C, Table 3). There was no significant association between reduction in LDL-C and study, recruitment centre, sex or genotype (Table 3).

**Table 3:** ANOVA—General linear model for the variation in LDL-C

Source	DF	Seq SS	Adj SS	Adj MS	F	*p*
Broccoli glucoraphanin phenotype a	1	566.0	527.3	527.3	4.78	0.031
Study	1	25.8	16.4	16.4	0.15	0.701
Recruitment centre	1	6.4	12.7	12.7	0.12	0.735
Sex	1	124.7	60.1	60.1	0.55	0.462
APOE genotype	5	219.8	168.2	33.6	0.30	0.909
PAPOLG genotype	2	117.4	123.0	61.5	0.56	0.574
GSTM1 genotype	1	22.4	22.4	22.4	0.20	0.653
Error	107	11802.4	11802.4	110.3		
Total	119	12884.9				

a HG, high glucoraphainin broccoli is heterozygous for Myb28^villosa^ allele and a Myb28^broccoli^ allele. Standard glucoraphanin broccoli is homozygous for Myb28^broccoli^ alleles.

**Figure 2:**
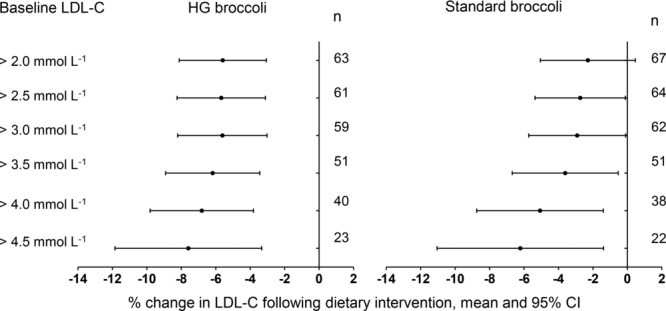
The mean and 95% CI for the change in LDL-C from paired *t*-tests between the baseline and postintervention LDL-C for each individual within the two dietary arms within subgroups with different baseline LDL-C. The data combines that of the current study with that of Armah et al. [2014].

The reduction in LDL-C by HG broccoli was not dependent upon baseline LDL levels (% ΔLDL-C = 5.8–2.6LDL-C_baseline_, *r*^2^ = 2.9%, *p* = 0.097), in contrast to that of standard broccoli that was significantly associated (% ΔLDL-C = 18.8–4.8LDL-C_baseline_, *r*^2^ = 13.7%, *p* = 0.001). Thus, once we reanalyse data according to subgroups of volunteers with different baseline LDL-C we observed a more moderate effect of standard broccoli at reducing LDL-C but only in volunteers who have higher baseline LDL-C (Fig. 2). There was some evidence, as expected, that HG broccoli would also reduce TC, but no evidence of any effect on HDL-C or TAG [Supporting Information Figs. 2–4]. Likewise, there was no evidence of an interaction between genotype and change in TC, HDL-C or TAG (Supporting Information Tables 2–4).

## 4 Discussion

Several previous human intervention studies with broccoli sprouts, *Brassica* extracts or mixed vegetable interventions that include crucifers have reported a reduction in LDL-C [1–5]. Furthermore, transfer of sheep from grazing grass to *Brassica* forage results in a reduction in plasma cholesterol [19]. While HG broccoli reduced plasma LDL-C to a greater extent that standard broccoli (Table 3), there is some indication that consuming standard broccoli may also be reducing plasma LDL-C, although in neither study 1 or 2, nor through combining the two studies is the reduction significantly different from baseline (*p* > 0.05). To ascertain the effect of standard broccoli on LDL-C would require a larger study. This however indicates that the reduction in LDL-C is due to the metabolic activity of glucoraphanin, as this is the major difference between the HG and standard broccoli.

The probable mechanism by which glucoraphanin reduces LDL-C is through the induction of nrf2-antioxidant response element mediated transcription by sulforaphane derived from glucoraphanin. Several animal studies suggest that nrf2 expression is closely associated with modulating mitochondrial fatty acid oxidation and lipid and steroid synthesis [20–23]. The two major signalling pathways that mediate cellular metabolism, PI3K signalling and AMP-activated protein kinase (AMPK) signalling, antagonistically regulate lipid and steroid biosynthesis and are both sensitive to regulation through the redox status of the cells and tissues. PI3K signalling enhances expression of sterol regulatory element-binding proteins (SREPBs) resulting in cholesterol synthesis [24–26], but is itself negatively regulated by phosphatase and tensin homolog (PTEN). In a more oxidising cellular environment, PTEN becomes inactivated, but is reactivated following disulphide bond reduction mediated by thioredoxin [27–31]. Thus, enhanced activity of thioredoxin reductase and supply of NADPH via the pentose phosphate pathway resulting from enhanced nrf2 mediated transcription may lead to activation of PTEN and concomitant down regulation of PI3K signalling and lipid and sterol synthesis. AMPK activity, in contrast, suppresses cholesterol biosynthesis through inhibition of acetyl-CoA carboxylase and 3-hydroxy-3-methylglutaryl-CoA reductase, and enhances fatty acid β oxidation [32]. However, AMPK is also redox sensitive and its activity is inhibited in a more oxidising cellular environment through oxidation and intermolecular disulphide bond formation, but is reactivated, in a similar manner to PTEN, by thioredoxin [33]. Thus, it is possible that diets rich in HG broccoli are able to reduce plasma LDL-C through modulating the redox status of cells and tissues resulting in enhanced activity of AMPK signalling and suppression of PI3K signalling.

More extreme forms of the phenotypic effect of nrf2 induction on lipid and cholesterol synthesis are reported in animal model systems in which the nrf2 inducers sulforaphane, oltipraz and the synthetic triterpenoid CDDO-imidazolide have each been shown to prevent obesity in mice fed high fat diets [20,34,35], an effect that is reduced in nrf2 knockout mice [34]. Furthermore, constitutive overexpression of the nrf2 ortholog SKN-1 prevents fat accumulation in *Caenorhabditis elegans* when fed a high carbohydrate diet [36].

The inhibition of cholesterol synthesis via nrf2-mediated redox regulation of signalling pathways would be consistent with the putative anticarcinogenic activity of cruciferous vegetables, in which maintenance of PTEN and AMPK activity would be expected to suppress cell proliferation and carcinogenesis [37].

*Brassica* vegetables are also dietary sources of S-methylcysteine sulfoxide (SMCSO) [38] that has been shown to reduce plasma LDL-C levels when administered to rodents [39,40]. However, the level of SMCSO in HG broccoli is approximately 20% less than in standard broccoli [9], and thus unlikely to explain the greater ability of the HG broccoli to reduce LDL-C compared to standard broccoli, although may still contribute to reducing plasma LDL-C levels by both broccoli genotypes. Likewise, there is no difference in the level of fibre between the broccoli genotypes [9], which may also contribute to reducing LDL-C levels. Other compounds, such as phytosterols may also have a role in reducing cholesterol, but due to the very limited introgression of the *B. villosa* genome into the broccoli genome [9], are unlikely to vary between the contrasting Myb28 genotypes.

It is also noteworthy that the reductions in LDL-C were due to consumption of broccoli that had been blanched and frozen. The blanching process destroys all endogenous plant thioglucosidase [‘myrosinase’] activity that is required to convert glucoraphanin to the nfr2 inducer sulforaphane. Several studies have reported that this conversion can also occur due to the activity of the gut microbiota [41,42], but with much less efficiency than if plant myrosinase is active. Thus, it is possible that consuming cooked fresh broccoli that retains a small myrosinase activity that can generate higher amounts of sulforaphane in the GI tract compared to gut micobiota-derived isothiocyanates [43], may have a more pronounced effect on reducing LDL-C than that reported in the current study.

There was a significant association between *APOE* genotype with baseline LDL-C, with higher levels associated with the E4 allele and lower levels with the E2 allele, consistent with previous reports (Table 2) [13,44]. However, there was no evidence of an interaction between *APOE* genotype and diet on LDL-C levels (Table 3), as has also been previously reported for the effect of dietary fat on LDL-C [13], or interactions with *GSTM1* and *PAPOLG* genotype (Table 3).

This proposed mode of action for LDL-C reduction contrasts with that of other known dietary components and pharmaceuticals that reduce LDL-C. Oat β glucans have been shown to reduce LDL-C by a similar extent to the HG broccoli described in the current study [45–47]. This reduction is likely to be due to reduced intestinal bile acid reabsorption leading to enhanced bile acid synthesis and a resultant reduction in plasma LDL-C [48]. Plant stanols have also been shown to reduce plasma LDL-C by between 7 and 10% [49]. These cholesterol-like molecules probably prevent uptake of cholesterol in the intestinal tract but may also interfere with cholesterol metabolism. Statins, which are now widely prescribed to reduce LDL-C, specifically inhibit 3-hydroxy-3-methylglutaryl-coenzyme A, a key enzyme in cholesterol synthesis. These drugs can reduce LDL-C by 20–45% depending upon dosage and the specific drug [50], with beneficial effects even for people who are at low risk of vascular disease [51].

In conclusion, evidence is provided that broccoli consumption may result in reduction in plasma LDL-C levels. This is likely to be due to a combination of dietary components found within broccoli, including glucoraphanin, fibre and SMCSO. HG broccoli reduces LDL-C to a greater extent than standard broccoli, and to a similar amount to that following intake of oat β glucans and plant stanols. This is likely to be due to the higher levels of glucoraphanin. The mechanism by which this reduction occurs is consistent with the inhibition of cholesterol synthesis as suggested by studies with animal models [20,22], as opposed to suppression of cholesterol or bile acid absorption. If this is the case, it is possible that combining broccoli with other dietary components that inhibit absorption may have an additive effect on the reduction of plasma LDL-C.

## Abbreviations

AMPK: AMP-activated protein kinase
APOE: apolipoprotein E
GSTM: glutathione transferase M1
HDL-C: high density lipoprotein cholesterol
HG: high glucoraphanin
LDL-C: low density lipoprotein cholesterol
Nrf2: nuclear factor [erythroid-derived 2]-like 2
PTEN: phosphatase and tensin homolog
PAPOLG: poly[A] polymerase gamma
SMCSO: *S*-methyl cysteine sulphoxide
TC: total cholesterol
TAG: triacylglycerol

## Supporting Information

As a service to our authors and readers, this journal provides supporting information supplied by the authors. Such materials are peer reviewed and may be re-organized for online delivery, but are not copy-edited or typeset. Technical support issues arising from supporting information (other than missing files) should be addressed to the authors.

Figure s1 Figure s2 Figure s3 Figure s4

**TableS1**.
