# Supplementary material for: Diet rich in high glucoraphanin broccoli reduces plasma LDL cholesterol: Evidence from randomised controlled trials
Source: Mol Nutr Food Res. 2015 Apr 7;59(5):918–26. doi: 10.1002/mnfr.201400863 (PMC4692095; doi:10.1002/mnfr.201400863)
Supplement: Supplementary file 1 [file mnfr0059-0918-sd1.pptx]

## Slide 1
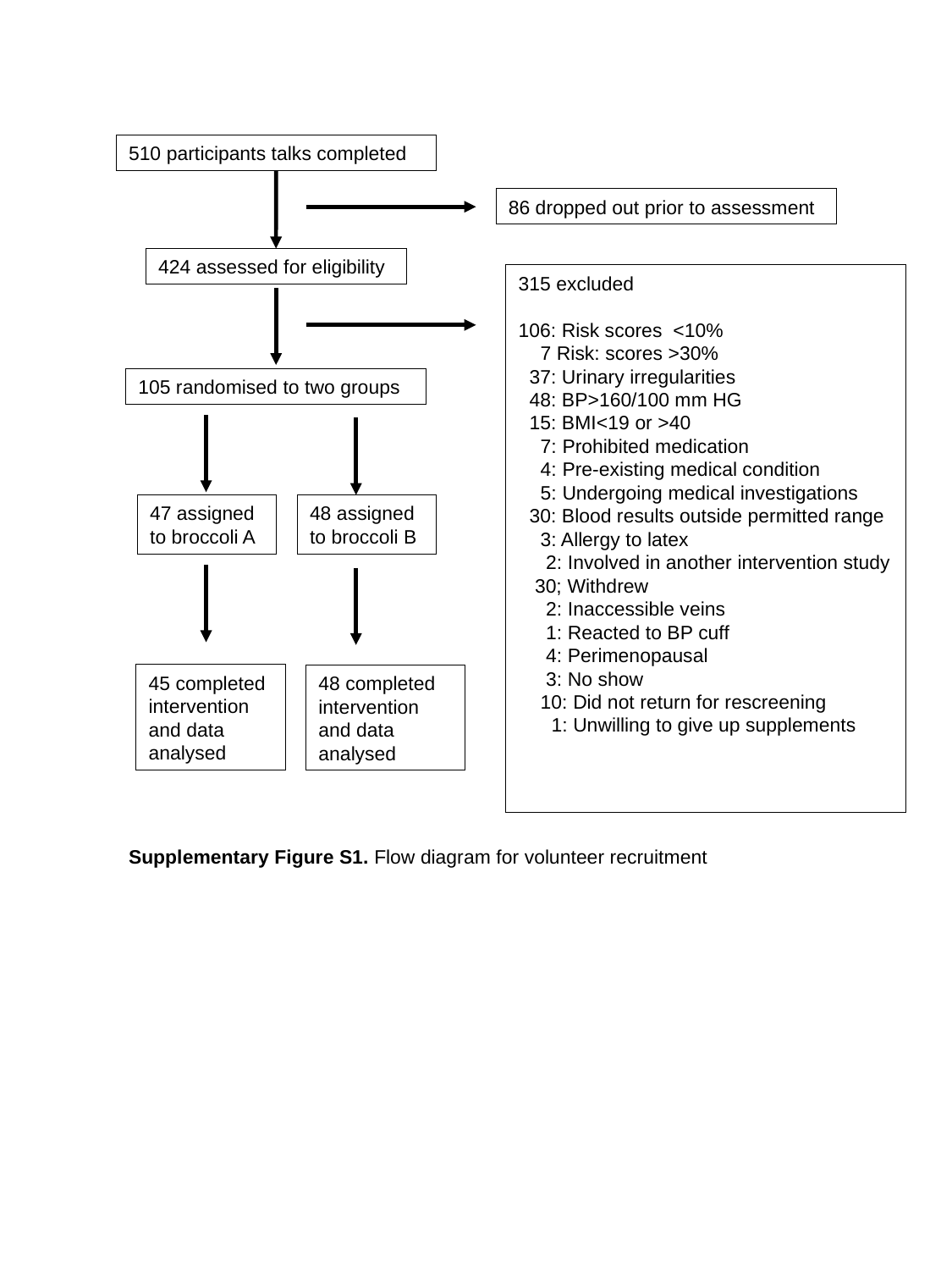

510 participants talks completed
86 dropped out prior to assessment
424 assessed for eligibility
315 excluded
106: Risk scores <10%
 7 Risk: scores >30%
 37: Urinary irregularities
 48: BP>160/100 mm HG
 15: BMI<19 or >40
 7: Prohibited medication
 4: Pre-existing medical condition
 5: Undergoing medical investigations
 30: Blood results outside permitted range
 3: Allergy to latex
 2: Involved in another intervention study
 30; Withdrew
 2: Inaccessible veins
 1: Reacted to BP cuff
 4: Perimenopausal
 3: No show
 10: Did not return for rescreening
 1: Unwilling to give up supplements
105 randomised to two groups
47 assigned to broccoli A
48 assigned to broccoli B
45 completed intervention and data analysed
48 completed intervention and data analysed
Supplementary Figure S1. Flow diagram for volunteer recruitment

## Slide 2
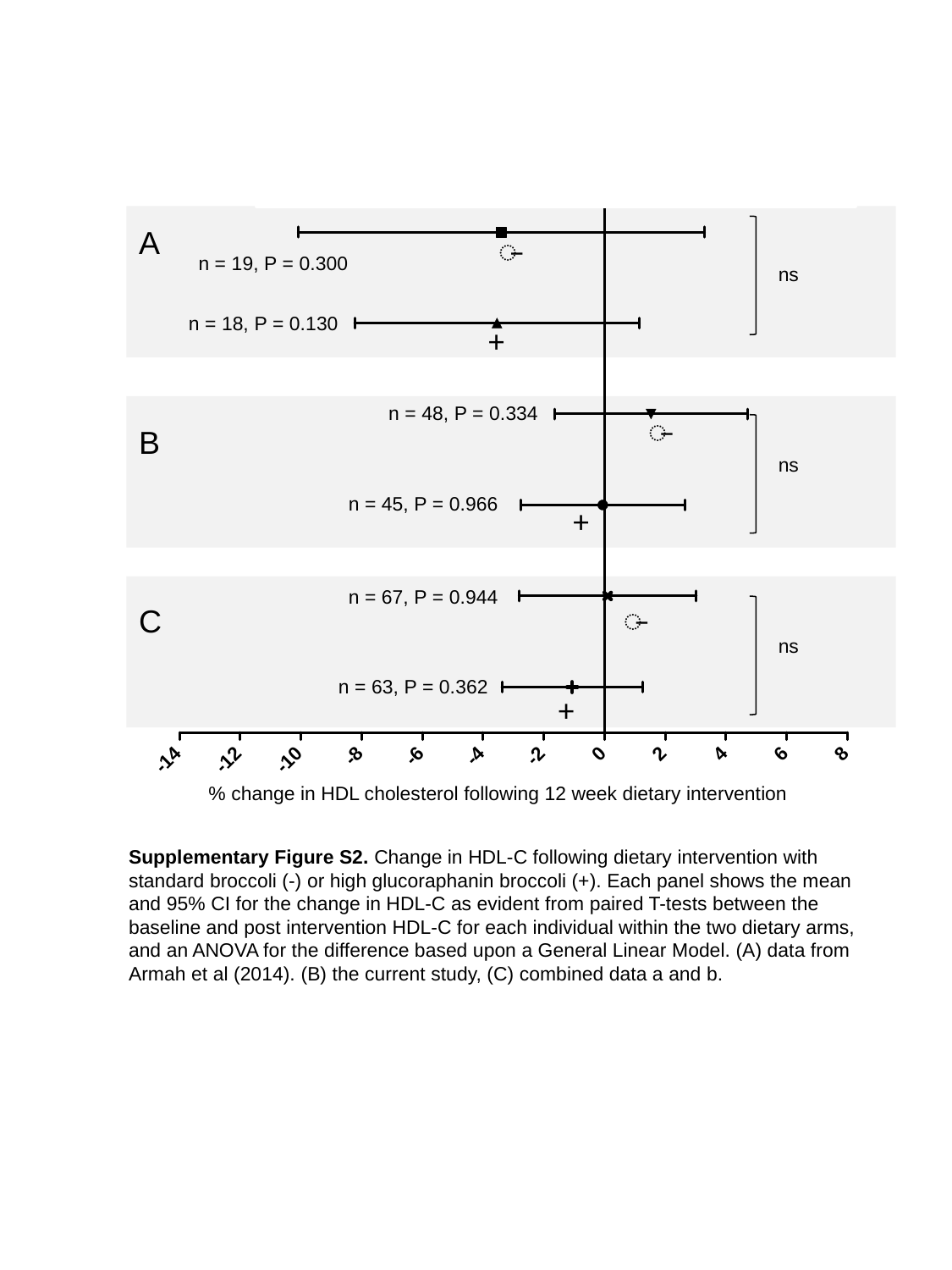

A
̶
n = 19, P = 0.300
ns
n = 18, P = 0.130
+
n = 48, P = 0.334
̶
B
ns
n = 45, P = 0.966
+
n = 67, P = 0.944
̶
C
ns
n = 63, P = 0.362
+
% change in HDL cholesterol following 12 week dietary intervention
Supplementary Figure S2. Change in HDL-C following dietary intervention with standard broccoli (-) or high glucoraphanin broccoli (+). Each panel shows the mean and 95% CI for the change in HDL-C as evident from paired T-tests between the baseline and post intervention HDL-C for each individual within the two dietary arms, and an ANOVA for the difference based upon a General Linear Model. (A) data from Armah et al (2014). (B) the current study, (C) combined data a and b.

## Slide 3
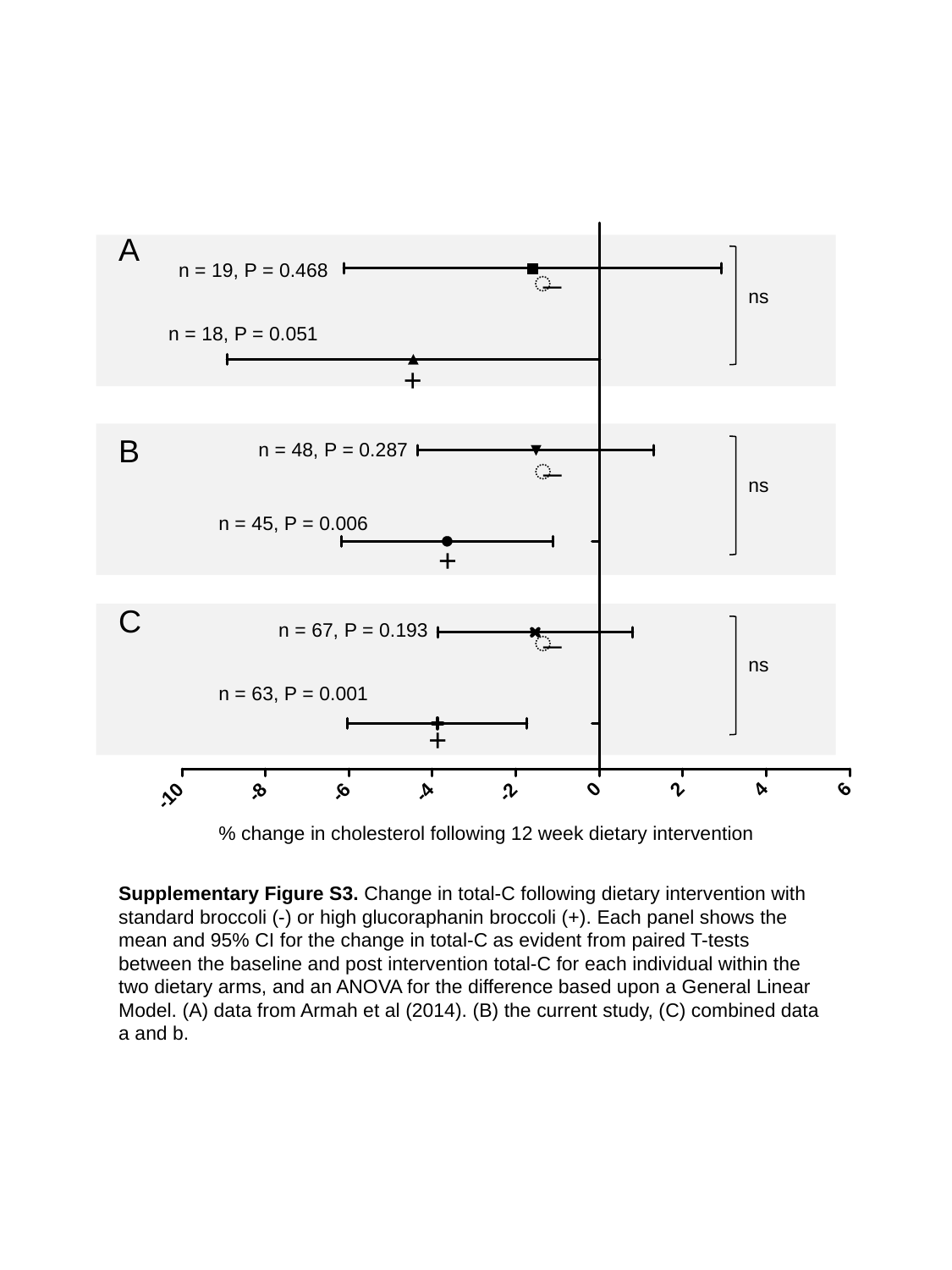

A
n = 19, P = 0.468
̶
ns
n = 18, P = 0.051
+
B
n = 48, P = 0.287
̶
ns
n = 45, P = 0.006
+
C
n = 67, P = 0.193
̶
ns
n = 63, P = 0.001
+
% change in cholesterol following 12 week dietary intervention
Supplementary Figure S3. Change in total-C following dietary intervention with standard broccoli (-) or high glucoraphanin broccoli (+). Each panel shows the mean and 95% CI for the change in total-C as evident from paired T-tests between the baseline and post intervention total-C for each individual within the two dietary arms, and an ANOVA for the difference based upon a General Linear Model. (A) data from Armah et al (2014). (B) the current study, (C) combined data a and b.

## Slide 4
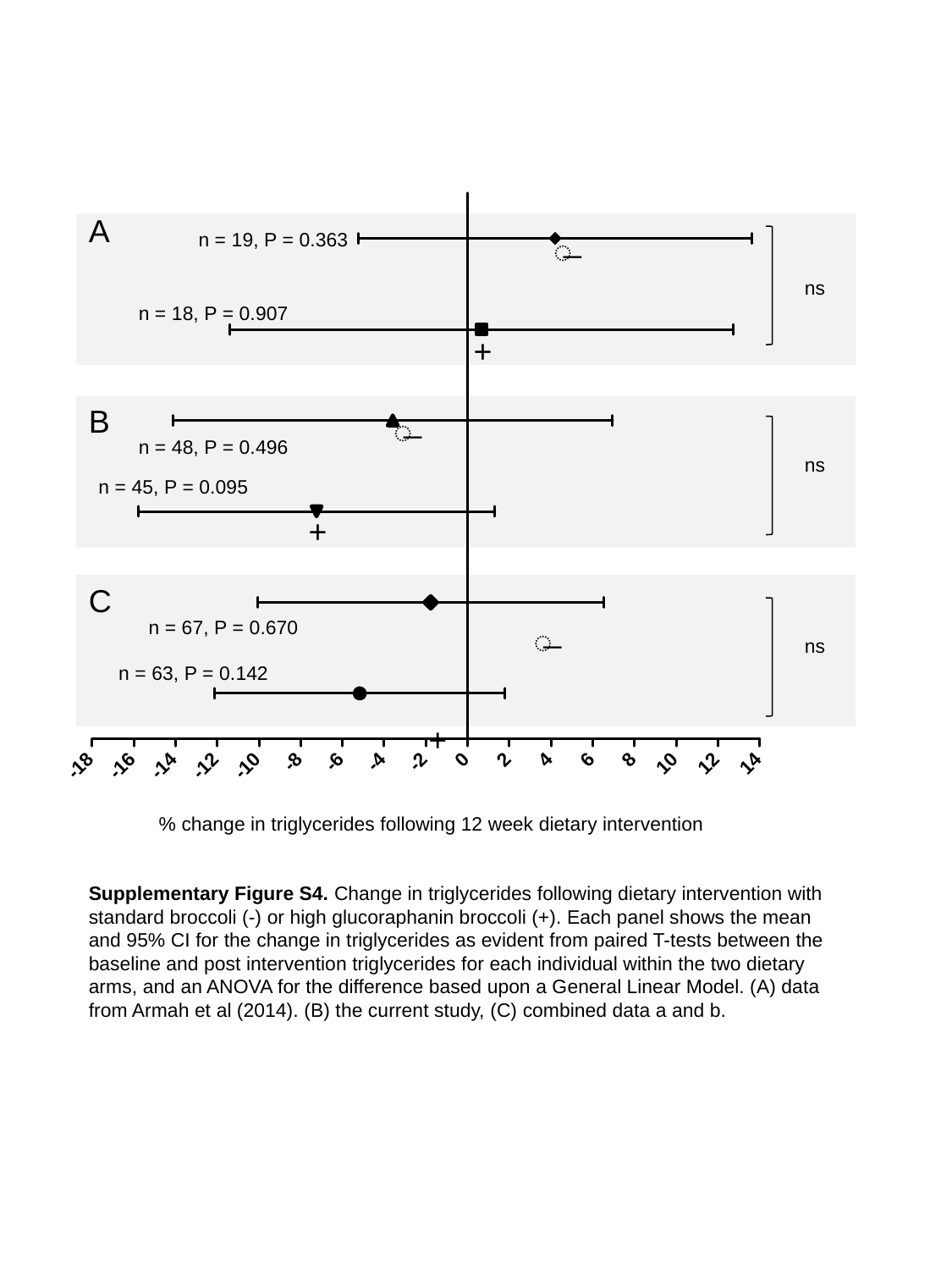

A
n = 19, P = 0.363
̶
ns
n = 18, P = 0.907
+
B
̶
n = 48, P = 0.496
ns
n = 45, P = 0.095
+
C
n = 67, P = 0.670
̶
ns
n = 63, P = 0.142
+
% change in triglycerides following 12 week dietary intervention
Supplementary Figure S4. Change in triglycerides following dietary intervention with standard broccoli (-) or high glucoraphanin broccoli (+). Each panel shows the mean and 95% CI for the change in triglycerides as evident from paired T-tests between the baseline and post intervention triglycerides for each individual within the two dietary arms, and an ANOVA for the difference based upon a General Linear Model. (A) data from Armah et al (2014). (B) the current study, (C) combined data a and b.
