# Supplementary material for: Diet rich in high glucoraphanin broccoli reduces plasma LDL cholesterol: Evidence from randomised controlled trials
Source: Mol Nutr Food Res. 2015 Apr 7;59(5):918–26. doi: 10.1002/mnfr.201400863 (PMC4692095; doi:10.1002/mnfr.201400863)
Supplement: Supplementary file 2 — TableS1. [file mnfr0059-0918-sd2.docx]

**Supplementary Table 1**

Mean (± SD) nutrient intake for participants on the HG and standard broccoli at baseline and week 10 in Study 2.

|  | HG broccoli (n=39) | |  | Standard broccoli (n=39) | |
| --- | --- | --- | --- | --- | --- |
|  | Baseline | Week 10 |  | Baseline | Week 10 |
| Energy (kcal) | 1946 ± 490 | 1967 ± 379 |  | 1840 ± 404 | 1874 ± 504 |
| Energy (MJ)  Fat (%E) | 8.1 ± 2.1  35 ± 4 | 8.2 ± 1.6  35 ± 5 |  | 7.7 ± 1.7  34 ± 5 | 7.8 ± 2.1  36 ± 6 |
| Protein (%E) | 16 ± 3 | 17 ± 3 |  | 17 ± 3 | 18 ± 4 |
| Carbohydrate (%E) | 46 ± 4 | 45 ± 5^1^ |  | 43 ± 6 | 42 ± 6^1^ |
| Total sugars (%E) | 20 ± 4 | 19 ± 4 |  | 19 ± 5 | 18 ± 6 |
| Starch (%E) | 26 ± 3 | 26 ± 4 |  | 24 ± 5 | 24 ± 5 |
| Cholesterol (mg) | 252 ± 76 | 271 ± 93 |  | 284 ± 72 | 314 ± 95 |
| NSP (Engl) (g) | 19 ± 5 | 19 ± 4 |  | 18 ± 5 | 18 ± 5 |
| Sodium (g) | 2.7 ± 1.0 | 2.7 ± 0.7 |  | 2.7 ± 0.8 | 2.6 ± 0.7 |
| Potassium (g) | 3.6 ± 0.8 | 3.6 ± 0.8 |  | 3.6 ± 1.0 | 3.5 ± 0.9 |
| Calcium (g) | 1.0 ± 0.3 | 1.0 ± 0.2 |  | 0.94 ± 0.3 | 0.9 ± 0.3 |
| Carotene (mg) | 3.3 ± 1.5 | 3.4 ± 1.7 |  | 4.4 ± 5.8 | 3.4 ± 1.3 |
| Vitamin D (μg) | 3.2 ± 1.8 | 3.0 ± 1.6 |  | 3.4 ± 1.5 | 3.3 ± 1.7 |
| Vitamin E (mg) | 10.4 ± 4.4 | 10.1 ± 3.6 |  | 10.4 ± 3.8 | 10.3 ± 3.6 |
| Vitamin C (mg) | 132 ± 51.8 | 169 ± 68 |  | 145 ± 81 | 145 ± 56 |
| Folate (μg) | 350 ± 95 | 369 ± 78 |  | 373 ± 139 | 363 ± 104 |

^1^ P=0.003 (T-test with Bonferroni correction for multiple comparions),

**Supplementary Table 2**

| Source | DF | Seq SS | Adj SS | Adj MS | F | P |
| --- | --- | --- | --- | --- | --- | --- |
| Broccoli genotype | 1 | 228.6 | 163.9 | 163.9 | 1.97 | 0.163 |
| Study | 1 | 7.95 | 5.87 | 5.97 | 0.07 | 0.791 |
| Recruitment centre | 1 | 0.20 | 10.8 | 10.8 | 0.13 | 0.719 |
| Sex | 1 | 91.8 | 39.9 | 39.9 | 0.48 | 0.490 |
| APOE genotype | 5 | 218.2 | 185.8 | 37.2 | 0.45 | 0.815 |
| PAPOLG genotype | 2 | 307.6 | 301.0 | 150.5 | 1.81 | 0.169 |
| GSTM1 genotype | 1 | 1.86 | 1.86 | 1.86 | 0.02 | 0.881 |
| Error | 108 | 8978.9 | 8978.9 | 83.1 |  |  |
| Total | 120 | 9835.2 |  |  |  |  |

ANOVA - General linear model for the change in Total cholesterol

**Supplementary Table 3**

ANOVA - General linear model for the variation in HDL-C

| Source | DF | Seq SS | Adj SS | Adj MS | F | P |
| --- | --- | --- | --- | --- | --- | --- |
| Broccoli genotype | 1 | 566.0 | 527.3 | 527.3 | 4.78 | 0.031 |
| Study | 1 | 25.8 | 16.4 | 16.4 | 0.15 | 0.701 |
| Recruitment centre | 1 | 6.4 | 12.7 | 12.7 | 0.12 | 0.735 |
| Sex | 1 | 124.7 | 60.1 | 60.1 | 0.55 | 0.462 |
| APOE genotype | 5 | 219.8 | 168.2 | 33.6 | 0.30 | 0.909 |
| PAPOLG genotype | 2 | 117.4 | 123.0 | 61.5 | 0.56 | 0.574 |
| GSTM1 genotype | 1 | 22.4 | 22.4 | 22.4 | 0.20 | 0.653 |
| Error | 107 | 11802.4 | 11802.4 | 110.3 |  |  |
| Total | 119 | 12884.9 |  |  |  |  |

**Supplementary Table 4**

ANOVA - General linear model for the variation in TAG

| Source | DF | Seq SS | Adj SS | Adj MS | F | P |
| --- | --- | --- | --- | --- | --- | --- |
| Broccoli genotype | 1 | 566.0 | 527.3 | 527.3 | 4.78 | 0.031 |
| Study | 1 | 25.8 | 16.4 | 16.4 | 0.15 | 0.701 |
| Recruitment centre | 1 | 6.4 | 12.7 | 12.7 | 0.12 | 0.735 |
| Sex | 1 | 124.7 | 60.1 | 60.1 | 0.55 | 0.462 |
| APOE genotype | 5 | 219.8 | 168.2 | 33.6 | 0.30 | 0.909 |
| PAPOLG genotype | 2 | 117.4 | 123.0 | 61.5 | 0.56 | 0.574 |
| GSTM1 genotype | 1 | 22.4 | 22.4 | 22.4 | 0.20 | 0.653 |
| Error | 107 | 11802.4 | 11802.4 | 110.3 |  |  |
| Total | 119 | 12884.9 |  |  |  |  |
